# Ethanol-induced transcriptional and translational changes in Aldh1l1-Egfp/Rpl10a cortical astrocyte cultures

**DOI:** 10.3389/fnins.2023.1193304

**Published:** 2023-06-21

**Authors:** Joel G. Hashimoto, Xiaolu Zhang, Marina Guizzetti

**Affiliations:** ^1^Department of Behavioral Neuroscience, Oregon Health and Science University, Portland, OR, United States; ^2^Research Service, VA Portland Health Care System, Portland, OR, United States

**Keywords:** astrocyte, FASD, translating RNA affinity purification (TRAP), alcohol, *in vitro*

## Abstract

The role astrocytes play in brain development and function has garnered greater attention as the diversity of roles they are involved in has become apparent. We have previously shown that ethanol-exposed astrocytes alter neuronal neurite outgrowth in an *in vitro* co-culture system and that ethanol alters the astrocyte-produced extracellular matrix (ECM) *in vitro*, with similar alterations *in vivo*. In this study, we utilized the translating ribosome affinity purification (TRAP) procedure in *Aldh1l1*-EGFP/Rpl10a transgenic mouse primary cortical astrocyte cultures to transcriptionally and translationally profile the astrocyte response to ethanol. We found a large number of differences between the total RNA pool and the translating RNA pool, indicating that the transcriptional state of astrocytes may not always reflect the translational state of astrocytes. In addition, there was a considerable overlap between ethanol-dysregulated genes in the total RNA pool and the translating RNA pool. Comparisons to published datasets indicate the *in vitro* model used here is most similar to PD1 or PD7 *in vivo* cortical astrocytes, and the ethanol-regulated genes showed a significant overlap with models of chronic ethanol exposure in astrocytes, a model of third-trimester ethanol exposure in the hippocampus and cerebellum, and an acute model of ethanol exposure in the hippocampus. These findings will further our understanding of the effects of ethanol on astrocyte gene expression and protein translation and how these changes may alter brain development and support the use of *in vitro* astrocyte cultures as models of neonatal astrocytes.

## Introduction

The significance of astrocytes as key players in brain development and function has been growing as the variety and number of crucial functions played by astrocytes have been discovered. Astrocytes are the main producers of the brain extracellular matrix (ECM), provide key structural and chemical cues for proper neuronal connections during brain development, stabilize and regulate synapses, and facilitate the creation and maintenance of the blood–brain barrier (Abbott et al., 2006; Santello et al., 2019; Kane and Drew, 2021), among other functions (Wilhelm and Guizzetti, 2016).

Recent advances in single-cell and single-nuclei RNA sequencing (RNA-Seq) and cell-type-specific expression profiling have further improved our understanding of the roles astrocytes play in the brain (Erickson et al., 2018; Brenner et al., 2020). However, studying astrocytes *in vivo* creates challenges based on the heterogeneity of the brain's cellular makeup and the complex feedback and cross-talk between different cell types. The use of *in vitro* models to study neurotoxic insults has provided a crucial understanding of the mechanistic underpinnings of complex diseases (Guttenplan et al., 2021; Kane and Drew, 2021). Mechanistic studies are particularly well suited to *in vitro* models, in which individual cell types can be manipulated in complex ways that would be untenable in whole tissues. However, one of the challenges associated with using *in vitro* models is interpreting how these models relate to *in vivo* processes.

Prenatal alcohol exposure (PAE) can lead to fetal alcohol spectrum disorders (FASD), resulting in brain and behavioral effects caused by a wide range of neurodevelopmental perturbations (Williams et al., 2015). PAE resulting in FASD impacts many different aspects of brain development and represents the largest type of preventable intellectual disability (Williams et al., 2015). Understanding the role of astrocytes in FASD-related brain dysfunction has been bolstered by *in vitro* studies in which astrocytes have been found to produce neuroinflammatory molecules, have altered extracellular protease expression, and have altered the production of chondroitin sulfate proteoglycans after ethanol exposure (Kane and Drew, 2021; Zhang et al., 2021; Goeke et al., 2022).

Gene expression studies utilizing high-throughput RNA-Seq typically analyze all RNA present in a given cell or tissue. Newer approaches have been developed to enrich cell-type-specific mRNA that is physically attached to the ribosome during the process of protein translation using the translating ribosome affinity-purification (TRAP) technology and the RiboTag system (Heiman et al., 2008; Sanz et al., 2009). These transgenic models result in cell-type-specific expression of tagged ribosomal proteins that, after incorporation into ribosomes, allow for the pull-down of cell-type-specific polysomes via immunoprecipitation methods and the assessment of mRNA abundance either by qRT-PCR or RNA-Seq. These approaches complement traditional RNA-Seq studies as the mRNA pool is enriched for specific cell types and actively translated, which is more closely linked to protein abundance (Heiman et al., 2014).

We have used both *in vitro* and *in vivo* methods to study the effects of developmental ethanol exposure. In our *in vivo* studies, we employed third trimester-equivalent human gestation models by exposing rats and mice to ethanol neonatally during the “brain growth spurt,” when astrocytes proliferate and provide crucial support and pathfinding cues to neurons (Goeke et al., 2018, 2022; Wilhelm et al., 2018; Zhang et al., 2021). To further understand how ethanol alters astrocyte functions during early brain development, we used the Aldh1l1-EGFP/Rpl10a transgenic mouse line, which allows for the analysis of astrocyte-specific mRNA translation. Here, we generated primary cortical astrocyte cultures from the Aldh1l1-EGFP/Rpl10a transgenic mouse line to assess translational and transcriptional changes caused by ethanol. Our data showed large differences in the total RNA vs. translating RNA pools.

However, the ethanol-induced alterations are very similar in terms of gene ontology (GO) and pathway enrichment analysis in the total RNA and translating RNA pools. A comparison of our datasets with published data indicates that our *in vitro* astrocytes were most similar to *in vivo* cortical astrocytes at postnatal days (PD) 1–7. In addition, broad similarities in the ethanol responses were observed in our datasets when compared to a neonatal model of ethanol exposure at PD10 in the hippocampus and the cerebellum, the hippocampus of PD7 mice administered a single dose of ethanol, and the medial prefrontal cortex (mPFC) following chronic ethanol exposure in adult mice. These comparisons provide context for our *in vitro* results on the adult and developmental impacts of ethanol exposure.

## Materials and methods

### Animals

Hemizygous B6;FVB-Tg(Aldh1l1-EGFP/Rpl10a)JD130Htz/J mice, originally generated by Nathaniel Heintz (Doyle et al., 2008), were obtained from The Jackson Laboratory (JAX, Bar Harbor, ME; Strain # 030247) and were bred with C57BL/6J mice to produce hemizygous offspring. Genotyping was carried out on a tail biopsy using a rapid DNA isolation protocol (Conner, 2002) and qPCR for eGFP and *Gapdh* for transgene presence and positive control, respectively, as previously described (Goeke et al., 2022). All animals were housed in the VA Portland Health Care System Veterinary Medical Unit on a 12-h light/dark cycle at an ambient temperature of 22 ± 1°C. All animal-related procedures were carried out in accordance with the National Institutes of Health Guidelines for the Care and Use of Laboratory Animals and were approved by the VA Portland Health Care System's Institutional Animal Care and Use Committee (Protocol # 4331).

### Primary astrocyte cultures and ethanol treatments

Primary mouse astrocyte cultures were created as previously described (Zhang et al., 2021; Goeke et al., 2022) using an equal number of transgenic male and female postnatal day (PD) 0 cortices. As the successful isolation and culturing of astrocytes are time-sensitive, the presence of the Aldh1l1-EGFP/Rpl10a transgene was detected using the Nightsea DFP-1 fluorescent protein flashlight and glasses in the dissected brain, with subsequent confirmation by qPCR for eGFP. In addition, sex determination was based on the anogenital distance and confirmed by qPCR for *Sry*, as previously described (Wilhelm et al., 2016). Astrocyte cultures were maintained in Dulbecco's Modified Eagle Medium (DMEM; Thermo Fisher Scientific, 11885092) with 10% fetal bovine serum (Atlanta Biologicals, S12450) and 1,000 U/ml penicillin-streptomycin (pen-strep, Thermo Fisher Scientific, 15140122) in a humidified incubator at 37°C under 5% CO_2_/95% air atmosphere for 14 days *in vitro* (DIV) to reach confluence. Astrocytes were subcultured onto 100 mm dishes at a density of 2.5 × 10^6^. After 6 days, the serum-containing medium was replaced with serum-free medium for 24 h, followed by 50 mM ethanol treatment in serum-free medium or control serum-free medium. To limit the effect of ethanol evaporation on culture conditions, dishes were placed in sealed chambers containing 5% CO_2_/95% air atmosphere with an open dish containing either 50 mM ethanol or water, as previously described by Goeke et al. (2018). After 24 h in the 50 mM ethanol or control medium, the medium was removed, and adherent astrocyte cells were processed for the TRAP procedure.

### Translating RNA affinity purification

TRAP was carried out on six control and six ethanol-treated 100 mm dishes, as previously described by Heiman et al. (2014) with modifications (Sanz et al., 2009). Briefly, homogenization buffer (HB) was used as described in Sanz et al., with heparin omitted. Lysed cells in HB were centrifuged at 10,000 g for 10 min at 4°C to remove insoluble debris, and the supernatant was used for all subsequent steps. Input samples, equivalent to 5% of the initial HB volume, were saved from the supernatant prior to the immunoprecipitation, and RNA was extracted at the same time as TRAP samples. This beginning clause is confusing 50 μg of two GFP antibodies (bioreactor supernatant, clones 19C8 and 19F7 from the Memorial Sloan-Kettering Institute Monoclonal Antibody Facility) was added to each sample for 4 h at 4°C under gentle end-over-end rotation. Samples were transferred to tubes containing 200 μl of Pierce Protein A/G Magnetic Beads (Thermo Fisher Scientific; 88803; lot VF298063) and incubated overnight at 4°C with end-over-end rotation. The following day, beads were washed in high-salt buffer as described in Sanz et al., and RNA was isolated from the beads and input samples using TRIzol (Thermo Fisher Scientific, 15596018) and the Direct-zol RNA Microprep Kit (Zymo Research, R2062). RNA concentration was determined using the Quant-iT RiboGreen RNA Assay (Thermo Fisher Scientific, R11490) using a CLARIOstar Plus plate reader (BMG Labtech). TRAP controls with either no antibody or using wild-type tissue result in no measurable RNA based on the RiboGreen assay and only trace amounts of RNA detected using qRT-PCR.

### RNA-Seq and analysis

TRAP (IP) samples from all 12 (six control and six ethanol) dishes and six input (three control and three ethanol) samples (selected arbitrarily) were submitted for RNA-sequencing at the OHSU Massively Parallel Sequencing Shared Resource. RNA quality was assessed using an Agilent Bioanalyzer, with all samples having a RIN of >8.8 (range 8.8–9.8). RNA-seq libraries were profiled on a 4200 Tapestation (Agilent) and quantified by real-time PCR using a commercial kit (Kapa Biosystems/Roche) on a StepOnePlus Real-Time PCR Workstation (ABI/Thermo). Libraries were then sequenced on a NovaSeq 6000 (Illumina). Fastq files were assembled from the base call files using bcl2fastq (Illumina). The fastq files were trimmed with Trimmomatic (Bolger et al., 2014; Bioinformatics 30, 2114) using the built-in filters for Illumina adapters. After trimming, sequence files were aligned with the STAR aligner (Dobin et al., 2013). The reference genome was Mus musculus GRCm38, downloaded with annotations from Ensembl. Following the alignment, the SAM files were converted to BAM format using SAMtools (Li et al., 2009). Short-read sequencing assays were performed using the OHSU Massively Parallel Sequencing Shared Resource (OHSU MPSSR). Raw read count data were analyzed using the R package DESeq2 (Love et al., 2014). Genes with less than a total of 10 reads across the 18 samples were excluded from the analysis. Clustering and principal component analysis were conducted on all 18 samples (inputs and IP), as well as an analysis of differential expression between the IP and input fractions. Differential expression/translation caused by ethanol treatment was carried out separately with each fraction. The significance of RNA-Seq data was determined using DESeq2 FDR-adjusted Wald test *p*-values of < 0.05. All processed and raw sequencing reads are publicly accessible at the Gene Expression Omnibus (GEO; https://www.ncbi.nlm.nih.gov/geo/) using accession number GSE227891.

### Validation of RNA-Seq by qRT-PCR

Confirmation of differential regulation by ethanol was carried out with qRT-PCR, as previously described by Goeke et al. (2022). Primers for mouse *Bcan* (Forward: 5′-CTGCGCGTCAAGGTAAACG-3′; Reverse: 5′-AGAGACACATCCGTGAGCGAT-3′), *Ncan* (Forward: 5′-GCTGGGGATCAGGACACAC-3′; Reverse: 5′-CAGTCTGAACCTTAGTCCACTTG-3′), and *Serpine1* (Forward: 5′-GCCACCGACTTCGGAGTAAA-3′; Reverse: 5′-TGAGCTGTGCCCTTCTCATT-3′) were used with 5 ng of RNA and the Luna Universal One-Step RT-qPCR Kit (NEB) with SYBR Green detection on the CFX96 Real-Time System (Bio-Rad). Cycle threshold data were normalized to total RNA using RiboGreen (ThermoFisher) and expressed as log_2_ transformed data relative to the input control samples, with significance determined by the Student's *t*-test.

### Bioinformatics analysis

Gene Ontology (GO) and pathway enrichment analysis was carried out using the enrichR package in R to query the enrichR database (Chen et al., 2013; Kuleshov et al., 2016). We limited the analysis to the following databases to focus our results on GO categories and known gene pathways: “GO_Molecular_Function_2021,” “GO_Cellular_Component_2021,” “GO_Biological_Process_2021,” “BioPlanet_2019,” “Elsevier_Pathway_Collection,” “KEGG_2021_Human,” “MSigDB_Hallmark_2020,” and “WikiPathway_2021_Human.” Significant gene categories were determined by unadjusted *p*-values < 0.01.

## Results

**Figure 1:**
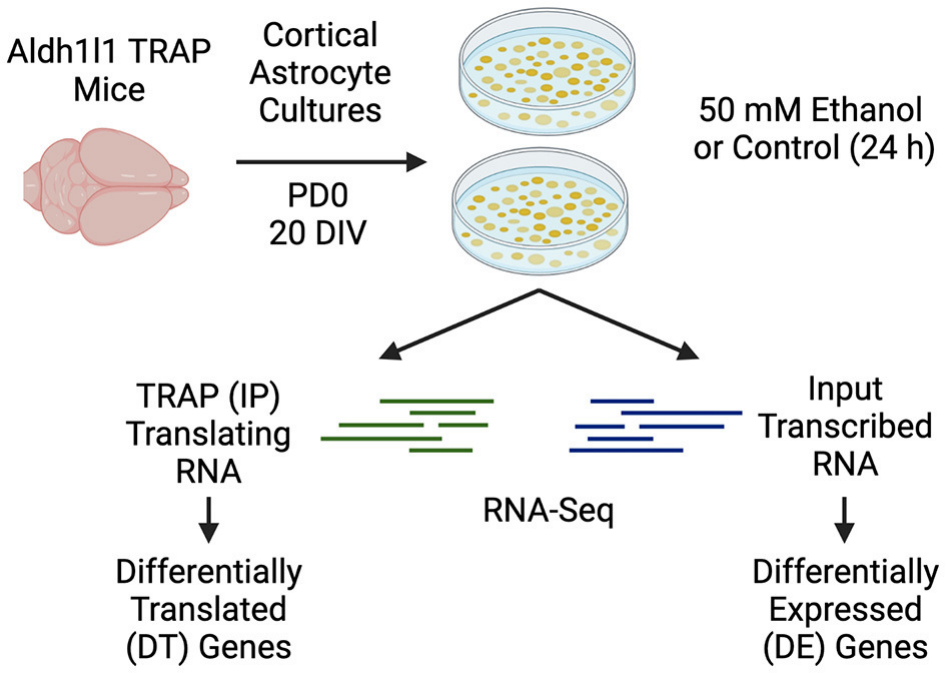
Experimental design. Primary cortical astrocyte cultures were generated from PD0 Aldh1l1-EGFP/Rpl10a transgenic mice. Astrocytes were cultured for 14 DIV before replating and were cultured for an additional 6 days. Serum was removed for 24 h prior to ethanol or control treatments for an additional 24 h. Following ethanol or control treatments, media was removed from the dishes, and the samples were processed for TRAP (IP), or input RNA, followed by RNA-Seq. Created with BioRender.com.

**Figure 2:**
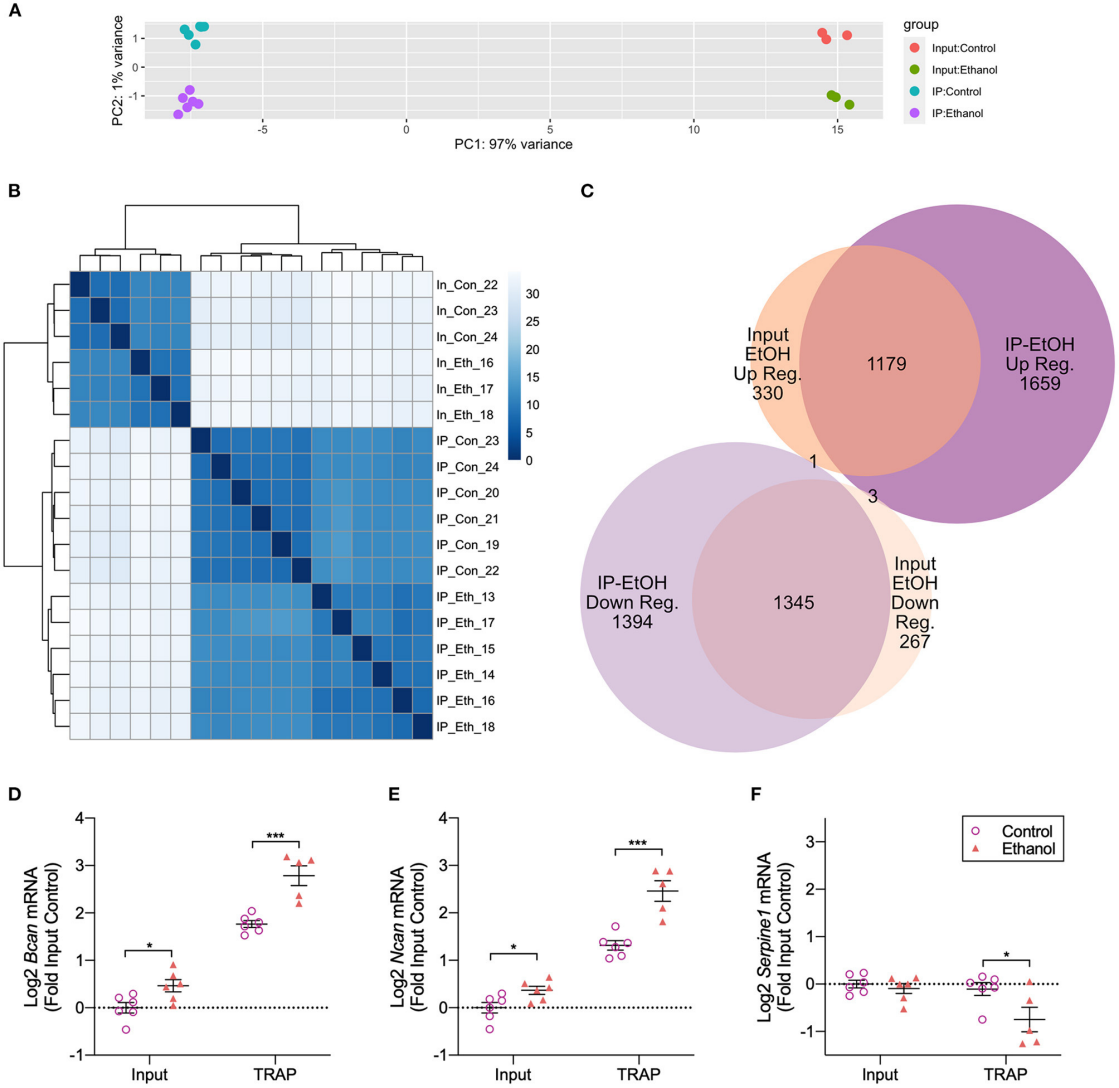
Translatome and transcriptome analyses of *in vitro* astrocytes following ethanol treatment. **(A)** Principal Component Analysis (PCA) of RNA-Seq data shows PC1 accounting for 97% of the variance separating IP and input samples. PC2 accounting for 1% of the variance separates control samples from ethanol-treated samples. **(B)** Hierarchical cluster analysis shows clear separation based on the fraction (input vs. IP) and treatment [control vs. ethanol (EtOH)]. **(C)** More genes were differentially regulated by ethanol in the IP fraction compared to the input fraction [5,581 (IP) vs. 3,125 (input)]. A comparison of ethanol-regulated genes in each fraction shows a large overlap between fractions, with 1,179 genes upregulated by ethanol in common between IP and input fractions and 1,345 genes in common between fractions that were downregulated by ethanol. **(D)** Confirmation of RNA-Seq results by qRT-PCR for *Bcan* showed increased *Bcan* mRNA in both input and TRAP fractions. The Log_2_ transformed data expressed relative to the input control with the mean ± SEM are shown. *p < 0.05; ***p < 0.001 (*n* = 5–6). **(E)** Confirmation of RNA-Seq results by qRT-PCR for *Ncan* showed increased *Ncan* mRNA in both input and TRAP fractions. The Log_2_ transformed data expressed relative to the input control with the mean ± SEM are shown. *p < 0.05; ***p < 0.001 (*n* = 5–6). **(F)** Confirmation of RNA-Seq results by qRT-PCR for *Serpine1*, which is the gene encoding PAI-1, showed decreased *Serpine1* mRNA in the TRAP fraction. The Log_2_ transformed data expressed relative to the input control with the mean ± SEM are shown. *p < 0.05 (*n* = 5–6).

To understand the differences in gene transcription and translation caused by ethanol treatments in primary astrocyte cultures, we performed the TRAP procedure on primary Aldh1l1-EGFP/Rpl10a astrocytes (Figure 1). The astrocytes were cultured for a total of 20 DIV, with a single pass at 14 DIV. Ethanol (50 mM) treatments were carried out in serum-free conditions, as we and others have done to model the *in vivo* environment of astrocytes (Prah et al., 2019; Zhang et al., 2021). An average of 103 million paired-end reads were obtained for each sample (standard deviation: 15.9 million, range 88.9–157.2 million), with a unique mapping average of 92.0% to the reference genome (range 89.7%−93.5%). Principle component analysis (PCA) of all RNA-Seq samples showed separation based on fraction (IP vs. input), which accounted for 97% of the variance (PC1), and ethanol treatment (PC2), which accounted for 1% of the variance (Figure 2A). Hierarchical clustering of all samples was conducted, resulting in the initial division of samples by fraction, followed by treatment (Figure 2B).

### Differential expression/translation analysis

Comparing IP vs. input fractions, we identified 12,313 (6,045 genes with higher read counts in IP vs. input and 6,268 genes with lower read counts in IP vs. input) genes using an adjusted (FDR) *p*-value cutoff of 0.05 (Supplementary Tables 1, 2). These results indicate that over half of the detected transcripts showed differential partitioning between the cytosol and the ribosome, as only transcripts being actively translated were pulled down during the TRAP procedure. The TRAP samples were from six independent dishes per treatment, while the RNA for the input samples was from aliquots taken from three of the control and three of the ethanol-treated IP (TRAP) samples; therefore, experimental variation due to differences in treatment conditions was minimized. The remaining three control and three ethanol-treated input samples were not sequenced due to design and cost considerations and were not excluded due to low yield or other technical issues.

Using DESeq2 to explore ethanol-induced differential expression/translation (DE and DT) within each fraction, we identified 5,581 DT genes in the TRAP (IP) fraction and 3,125 DE genes in the input fraction (Supplementary Tables 3, 4, respectively). A comparison of the direction of ethanol regulation between the fractions showed that 1,179 genes were upregulated by ethanol in the IP and input fractions, and 1,345 genes were downregulated in both the IP and input fractions. In addition, we identified 1,659 genes that were only upregulated by ethanol in the IP fraction and 1,394 genes that were only downregulated in the IP fraction. We also found 330 genes that were only upregulated by ethanol in the input fraction and 267 genes that were downregulated by ethanol in the input fraction (Figure 2C). Four genes were differentially regulated by ethanol in opposite directions based on the fraction, with three upregulated by ethanol in IP and downregulated by ethanol in input (*Chd3, Meg3*, and *Ndst1*) and one gene downregulated by ethanol in IP and upregulated by ethanol in input (*Sumo1*).

RNA-Seq results were validated by qRT-PCR utilizing the same samples used for RNA-Seq. We confirmed the upregulation of *Bcan* [input: *t*_(10)_ = 2.732, *p* = 0.021; TRAP: *t*_(9)_ = 4.982, *p* = 0.0008] and *Ncan* [input: *t*_(10)_ = 2.603, *p* = 0.026; TRAP: *t*_(9)_ = 5.133, *p* = 0.0006] by ethanol in both input and TRAP fractions (Figures 2D, E). In addition, we confirmed the downregulation of *Serpine1* [input: *t*_(10)_ = 0.7059, *p* = 0.496; TRAP: *t*_(9)_ = 2.325, *p* = 0.045] in the TRAP fraction by ethanol (Figure 2F). We have previously shown that brevican (encoded by the gene *Bcan*) and neurocan (encoded by the gene *Ncan*) protein levels are upregulated by ethanol and that plasminogen activator inhibitor 1 (PAI-1; encoded by the gene *Serpine1*) protein levels are downregulated by ethanol in astrocyte cultures measured by Western blot and/or ELISA (Wilhelm et al., 2018; Zhang et al., 2021), indicating that changes in translating RNA levels in our TRAP fraction result in corresponding changes in protein abundance.

Due to the differences in the number of samples processed between fractions and the large difference in the number of ethanol-regulated genes we identified in the IP vs. input fraction, we investigated how the difference in sample sizes impacted the number of ethanol-regulated genes. Because our samples are matched with IP samples immunoprecipitated from the lysate sampled for the input, we limited our analysis to the three control and three ethanol-treated samples with both input and IP RNA-Seq results. We re-ran the same data analysis pipeline with the limited IP data and found a similar number of ethanol-regulated genes in the limited data set of the IP fraction and in the input fraction (3,317 DT genes in the limited dataset compared to 3125 DE genes in the input samples). This indicates that most of the difference in the number of ethanol-regulated genes identified in each fraction was due to the higher number of samples in the IP comparison (Supplementary Table 5). For all subsequent analyses and discussions, we will refer to the analysis of the full complement of IP samples unless explicitly indicated.

### Bioinformatics analysis: gene category enrichment

**Figure 3:**
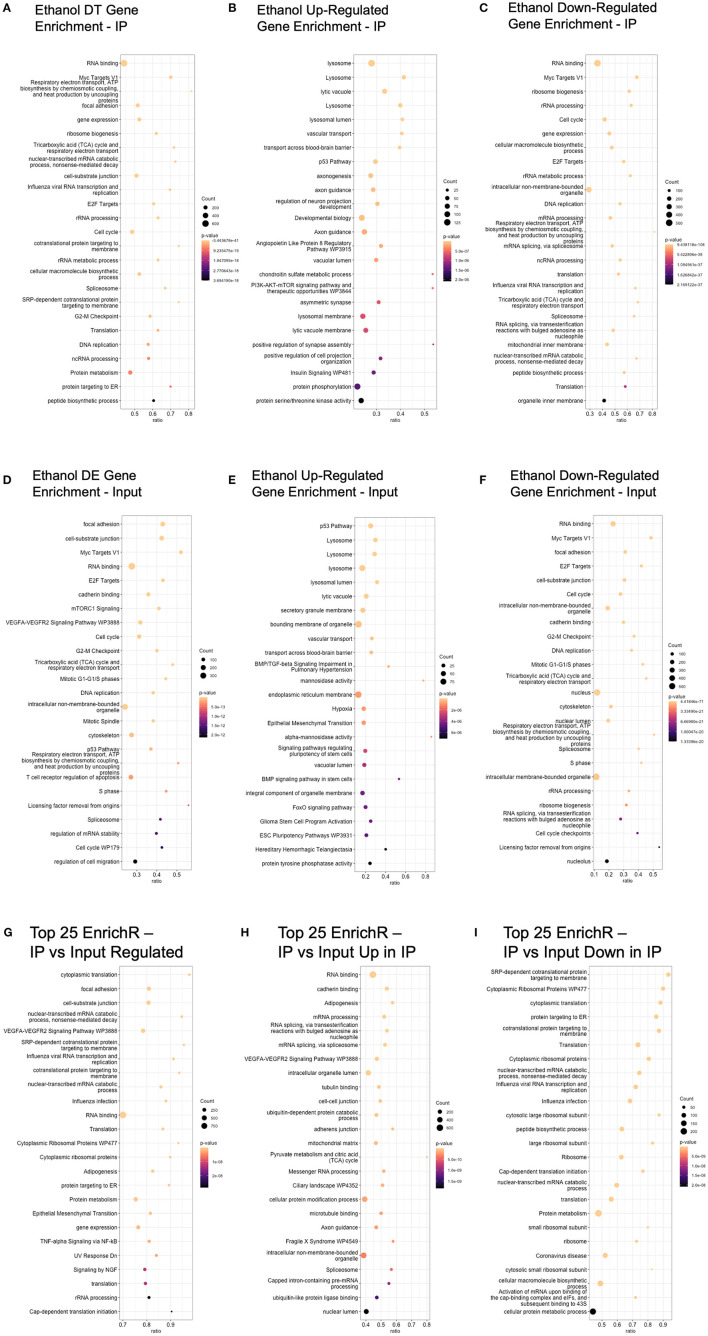
Top 25 enrichR results (based on enrichment *p*-value) for each comparison. **(A)** Ethanol DT genes in the IP fraction were analyzed using enrichR to identify enriched GO categories or pathways. **(B)** Ethanol-upregulated DT genes were used to query the enrichR database. **(C)** Ethanol-downregulated DT genes were used to query the enrichR database. **(D)** Ethanol DE genes in the input fraction were analyzed using enrichR to identify enriched GO categories or pathways. **(E)** Ethanol-upregulated DE genes in the input fraction were used to query the enrichR database. **(F)** Ethanol-downregulated DE genes in the input fraction were used to query the enrichR database. **(G)** Genes with different read counts between IP and input fractions were analyzed using enrichR to identify enriched GO categories and pathways. **(H)** Genes with higher read counts in IP compared to input were analyzed using enrichR to identify enriched GO categories and pathways. **(I)** Genes with higher read counts in input compared to IP were analyzed using enrichR to identify enriched GO categories and pathways.

Ethanol-regulated DE and DT genes were used to query the Enrichr database to identify GO and pathway enrichment (Chen et al., 2013; Xie et al., 2021). In the IP samples, we identified 1,111 categories as enriched at an unadjusted enrichment *p*-value of < 0.01. When sub-setting the genes based on the direction of ethanol regulation, we identified 711 and 1,120 significant gene categories in upregulated and downregulated genes, respectively (Supplementary Table 6). The top 25 categories in each comparison, based on *p*-value, are shown in Figures 3A–C. Of note, we identified several categories related to RNA and ribosomes (“RNA binding,” “ribosome biogenesis,” “rRNA processing”) in ethanol-regulated and ethanol-downregulated genes in the IP fraction. Of the 140 genes that were ethanol regulated in the “Myc Targets V1” category in the IP fraction, 135 were downregulated by ethanol, indicating that Myc may be a primary target of ethanol. Similarly, of the 17 genes in the “Chondroitin sulfate metabolic process,” which was highly significant in the ethanol-upregulated genes in the IP fraction (*p* = 5.26 × 10^−7^), and all ethanol-regulated genes (*p* = 8.69 × 10^−4^), 16 were upregulated by ethanol, suggesting that this process was highly upregulated in ethanol-exposed astrocytes. In addition, we identified 16 other glycosaminoglycan/chondroitin sulfate proteoglycan-related categories that were upregulated by ethanol in the IP fraction, which showed few to no genes in the ethanol-downregulated group in the IP fraction (Supplementary Table 6). Other categories with highly skewed makeup between upregulated and downregulated genes by ethanol include lysosome-related functions/cellular components, “transport across the blood–brain barrier,” and axonogenesis/axon guidance, with more genes upregulated than downregulated by ethanol.

In the input samples, we identified 1,142 enriched gene categories, with 657 and 1,277 gene categories in ethanol upregulated and downregulated genes, respectively (Figures 3D–F and Supplementary Table 6). Mirroring the results from the IP analysis, “Myc Targets V1,” “RNA binding,” and “cell cycle” were significant only in the ethanol-downregulated genes in the input fraction. Lysosome-related categories (“lysosome,” “lysosomal lumen”), “transport across the blood–brain barrier,” and “vascular transport” were significant only in the ethanol-upregulated genes in the input fraction.

Comparing these analyses, we found that only 15 categories were significant in all of the analyses of genes upregulated, downregulated, and regulated in either direction in the IP fraction, indicating that these 15 pathways were impacted irrespective of the direction of ethanol regulation (Supplementary Table 7). The categories include the “androgen receptor signaling pathway,” the “integrin signaling pathway,” and “BDNF signaling pathway.” In the input fraction, we found 59 significant categories for genes that were upregulated, downregulated, and ethanol regulated in either direction (Supplementary Table 7). The categories with significant enrichment in up, down, and both directions in the input fraction include “focal adhesion,” “TGF-beta signaling pathway,” and “cell-substrate junction.” The four categories were enriched in all six comparisons across IP, and input fractions (ethanol-regulated IP, ethanol-upregulated IP, ethanol-downregulated IP, ethanol-regulated input, ethanol-upregulated input, ethanol-downregulated input) were “PI3/AKT/mTOR signaling,” “BDNF signaling pathway,” “UV Response Up,” and “Apical Junction.”

A comparison of the significant GO and pathway categories identified in ethanol-regulated genes in the IP and input fractions shows 732 categories as significant in both the input and IP fractions. The high proportion (66 and 64% in IP and input, respectively) of gene category similarity is expected based on the high number of overlapping regulated genes in each fraction.

We also conducted pathway and GO enrichment analysis on the genes that were identified as significantly different between the input and IP fractions, with the top 25 categories enriched in the analysis of upregulated and downregulated genes, upregulated in IP, and upregulated in input shown in Figures 3G–I, respectively. Enriched categories, irrespective of direction, included “cytoplasmic translation,” “cell-substrate junction,” “VEGFA-VEGFR2 Signaling Pathway,” “cotranslational protein targeting to the membrane,” and “RNA binding.” Top categories in genes that were higher in the IP fraction compared to the input that were not also significant in genes that were higher in the input fraction compared to IP (which can be characterized as gene categories that are actively translated) included “RNA binding,” “cadherin binding,” “VEGFA-VEGFR2 Signaling Pathway,” and “Axon guidance.” Top categories in genes that were higher in the input fraction compared to the IP fraction that were not also significant in genes that were higher in the IP fraction compared to input (which can be characterized as gene categories related to RNAs that are present in the cell but not actively translated) included “SRP-dependent cotranslational protein targeting to membrane,” “Cytoplasmic Ribosomal Proteins,” and “Translation.”

### Comparison to developmental astrocyte TRAP studies

**Figure 4:**
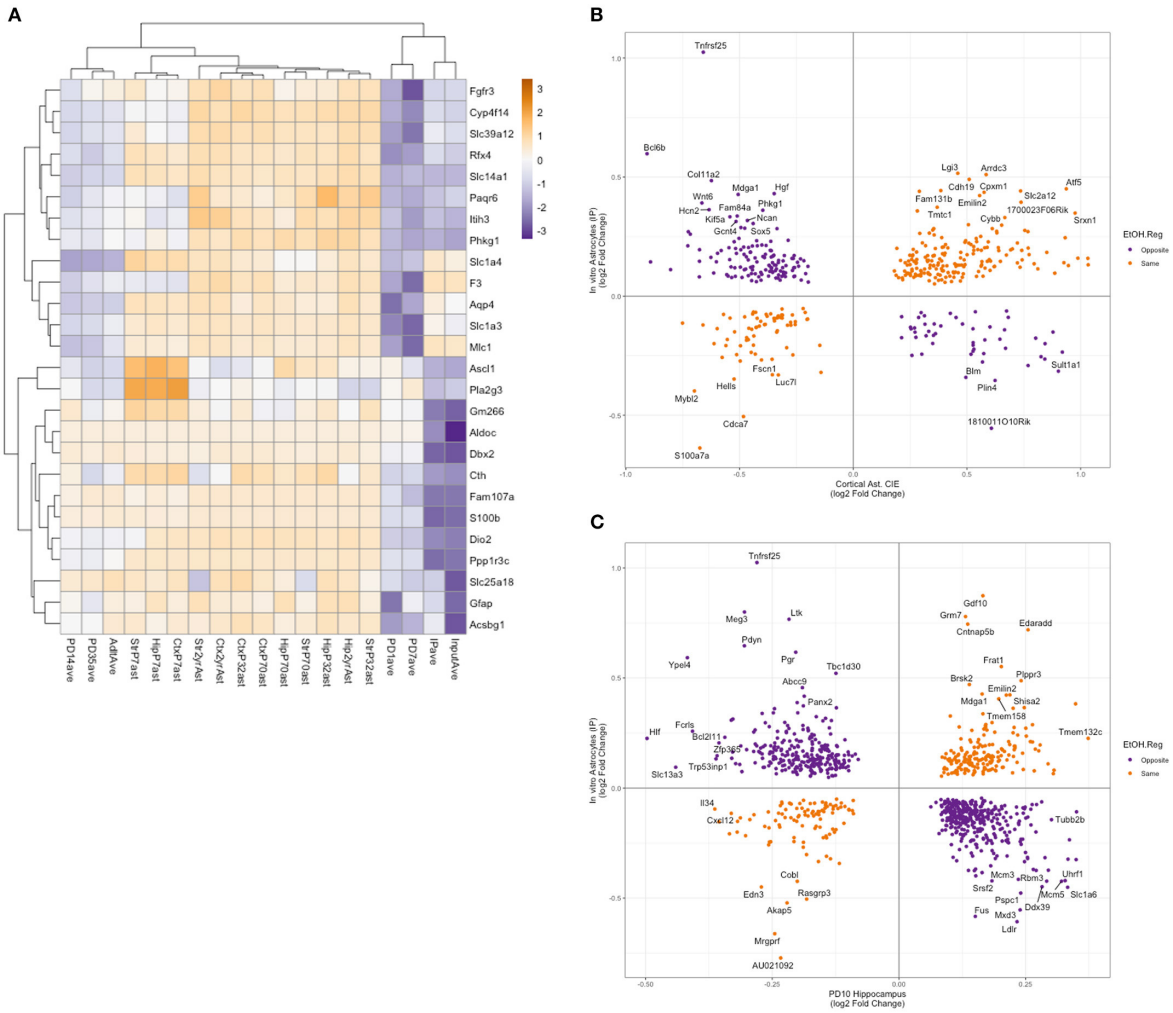
Comparison of *in vitro* astrocyte TRAP RNA-Seq to published datasets of *in vivo* astrocyte expression. **(A)** Astrocyte-enriched genes identified as altered during development (as in Rurak et al., 2022) were clustered using the percentile rank of each gene to account for differences between the experiments (Clarke et al., 2018; Rurak et al., 2022). The clustering of these genes indicates that our *in vitro* RNA-Seq expression is more similar to PD1 and PD7 cortical astrocyte expression. **(B)** An overlap of ethanol-regulated genes in *in vivo* astrocytes after chronic intermittent ethanol exposure and DT genes in the IP fraction from *in vitro* astrocytes. Genes with the same direction of regulation by ethanol are shown in orange, and genes with the opposite direction of regulation by ethanol are shown in purple. Genes with a log_2_ fold change greater than abs 0.3 are labeled with the gene symbol. **(C)** An overlap of ethanol-regulated genes in PD10 hippocampus after ethanol exposure and DT genes in the IP fraction from *in vitro* astrocytes. Genes with the same direction of regulation by ethanol are shown in orange, and genes with the opposite direction of regulation by ethanol are shown in purple. Genes with a log_2_ fold-change greater than abs 0.35 in the PD10 hippocampus or abs 0.4 in the *in vitro* astrocytes are labeled with the gene symbol.

To better understand the relevance of *in vitro* astrocyte transcription and translation to the *in vivo* setting, we compared our results to previously published studies on the Aldh1l1-EGFP/Rpl10a mice. Clarke et al. (2018) profiled astrocyte translation in the striatum, hippocampus, and cortex at PD7, PD32, 10 weeks, 9.5 months, and 2 years using TRAP, followed by RNA-Seq. Rurak et al. (2022) profiled the cortex in males and females at PD1, PD4, PD7, PD14, PD35, and adult time points using TRAP followed by RNA-Seq. To observe what developmental stage and brain region(s) our *in vitro* astrocytes are most similar to in *in vivo* astrocytes, we conducted a hierarchical cluster analysis on 26 genes identified as being astroglial-specific and developmentally regulated (Rurak et al., 2022). As our data were from mixed-sex cultures, we collapsed sex data based on time points for the Rurak et al. analysis. In addition, to account for differences in library preparation and sequencing protocols, we calculated the average percentile for each gene in each age and tissue. Inspection of the cluster dendrogram showed that our *in vitro* astrocytes (both input and IP fractions) cluster most closely with the PD1 and PD7 cortical astrocytes (Figure 4).

### Comparison to chronic intermittent ethanol effects on *in vivo* astrocytes

We then compared the genes that were identified as DE or DT in our input and IP fractions to an analysis of astrocytes isolated via magnetic bead/antibody complexes targeting an astrocyte-specific surface marker (*Acsa2*) from mice that had undergone chronic intermittent ethanol exposure (CIE). The CIE model is well established in the literature as a model of binge exposure, which can cause increased drinking and dependence. Erickson et al. (2018) identified 1,153 differentially expressed genes in astrocytes after CIE. We compared our DT and DE genes to these genes regulated *in vivo* and saw 434 and 253 genes in common, respectively (Figures 4B, C). Hypergeometric analysis, using the R package GeneOverlap (Shen, 2022), showed a significant overlap between the *in vivo* astrocytes and both fractions (IP: *p* = 1.8 × 10^−17^, input: *p* = 2.9 × 10^−11^), indicating that the similarity of the gene sets was unlikely to be random. As observed in Figure 4B, there was a significant proportion of the genes that were regulated by ethanol in the opposite direction between the *in vivo* and *in vitro* astrocytes, and hypergeometric testing of the genes based on the direction of regulation did not show a significant overlap. An analysis of GO and pathway enrichment of the ethanol-regulated genes in common between the *in vivo* and *in vitro* data showed categories related to the lysosome, pre- and post-synapse, and extracellular matrix, which in many ways is consistent with what we observed in the GO and pathway enrichment analysis of DT and DE genes (Figure 3 and Supplementary Table 8). While conducting the enrichment analysis on genes that were regulated in opposite directions between *in vitro* and *in vivo* datasets, gene categories that potentially relate to the cross-talk between astrocytes and other brain cell types or the extracellular matrix were overrepresented, with “regulation of synapse assembly,” “positive regulation of cell junction assembly,” and “nervous system development” enriched in IP genes regulated in the opposite direction from *in vivo* astrocytes, and “regulation of cell migration,” “Dermatan sulfate biosynthesis,” and “extracellular matrix organization” enriched in input genes regulated in the opposite direction from *in vivo* astrocytes (Supplementary Table 8).

### Comparison to neonatal ethanol-treated mice

We also sought to compare our DT and DE genes with a neonatal model of third-trimester equivalent ethanol exposure in mice. Key developmental processes that occur in the third trimester of human gestation, occur in the first two post-natal weeks in mice (Clancy et al., 2001). Pinson et al. (2021) administered 4 g/kg of ethanol from PD4-PD9 and examined ethanol-induced changes in hippocampal and cerebellar gene expression at PD10 24 h after the last dose of ethanol administered using RNA-Seq. After neonatal ethanol exposure, they found 2,150 DE genes in the hippocampus and 2,017 DE genes in the cerebellum (Pinson et al., 2021). Comparison of our DT and DE genes from *in vitro* astrocytes showed an overlap of 903 genes in common between DT (IP) and PD10 DE genes from the hippocampus, 596 genes in common between DE (input) and PD10 DE genes from the hippocampus, 777 genes in common between DT (IP) and PD10 DE genes from the cerebellum, and 497 genes in common between DE (input) and PD10 DE genes from the cerebellum (Supplementary Table 9). Hypergeometric analysis showed a significant overlap in all four comparisons (IP vs. hippocampus: *p* = 1.5 × 10^−60^, input vs. hippocampus: *p* = 5.6 × 10^−60^; IP vs. cerebellum: *p* = 3.5 × 10^−35^, input vs. cerebellum: *p* = 4.1 × 10^−34^). GO and pathway enrichment analysis showed enriched categories relating to “RNA binding,” “Myc Targets V1,” and “Protein metabolism” in the overlapping genes between both the IP and input ethanol-regulated genes in our analyses and the PD10 hippocampus (Supplementary Table 10). GO and pathway enrichment analysis of overlapping genes between the IP and input ethanol-regulated genes from our analysis with the PD10 cerebellum ethanol-regulated genes showed enrichment in categories relating to “cytoplasmic translation,” “cytoplasmic ribosomal proteins,” and “SRP-dependent cotranslational protein targeting to the membrane.” While examining the direction of regulation in the overlapping genes, we observed that a higher number of genes were regulated by ethanol in opposite directions between *in vitro* astrocytes and the whole hippocampus or cerebellum [IP vs. hippocampus: 283 (same), 620 (opposite); input vs. hippocampus: 155 (same), 441 (opposite); IP vs. cerebellum: 232 (same), 545 (opposite); input vs. cerebellum: 145 (same), 352 (opposite)]. Moreover, hypergeometric analysis of the overlap of genes regulated by ethanol in the same direction showed no significant overlap. This is likely due to many factors, including the difference between *in vivo* and *in vitro* ethanol treatments (with a major difference being that in the study by Pinson et al., tissue samples were collected 24 h after the last administration of ethanol, corresponding to an alcohol withdrawal time point, while in our experiments, samples were collected immediately after alcohol exposure), the difference in brain areas (*in vitro* astrocytes were generated from the cortex while the *in vivo* study examined the hippocampus and the cerebellum), and the presence of multiple cell types in the whole tissue expression profiles. GO and pathway enrichment analyses of overlapping genes in the same direction in IP and the hippocampus showed pathways related to “cell-cell adhesion via plasma-membrane adhesion molecules,” “proteoglycans in cancer,” and “axon guidance,” while overlapping genes in the same direction in IP and the cerebellum showed pathways related to “wound healing,” “positive regulation of cell migration,” and “signaling events mediated by focal adhesion kinase.” GO and pathway enrichment analysis of genes that were regulated in opposite directions between IP and the hippocampus returned categories “RNA binding,” “Myc Targets V1,” and “protein metabolism,” while oppositely regulated genes between IP and the cerebellum returned categories “cytoplasmic ribosomal proteins,” “cytoplasmic translation,” and “cotranslational protein targeting the membrane.”

As our *in vitro* exposure is a single 24 h exposure, we wanted to compare our data to a recently published dataset from C57BL/6J mice that were administered a single dose of ethanol on PD7 (Baker et al., 2022). Baker et al. (2022) used a microarray platform to identify ethanol-regulated genes in males and females of several different recombinant inbred BXD lines and the parental C57BL/6J (B6) and DBA/2J lines. A comparison of the ethanol-regulated genes from our *in vitro* astrocytes to the ethanol-regulated genes in the B6 hippocampus from either males or females shows a large number of genes in common, with 158 genes in the IP fraction and 113 genes in the input fraction. Hypergeometric analysis shows a significant overlap in both comparisons (IP vs. B6: 1.2 × 10^−20^; input vs. B6: 2.4 × 10^−21^). Interestingly, in this comparison, we observed that the vast majority of the ethanol regulation *in vitro* and in the hippocampus after a single ethanol exposure was in the same direction. We found 142 genes regulated in the same direction in the IP fraction (hypergeometric overlap: *p* = 1.4 × 10^−13^), with only 16 genes regulated in the opposite direction, and 104 genes regulated in the same direction in the input fraction (hypergeometric overlap: *p* = 8.1^−17^), while only nine were regulated in opposite directions (Supplementary Table 11). This suggests that the exposure model used in our *in vitro* studies more closely resembled a single exposure *in vivo*.

## Discussion

In this study, we utilized the TRAP procedure to generate transcriptional (input) and translational (IP) profiles of Aldh1l1-EGFP/Rpl10a primary cortical astrocyte cultures following ethanol treatment that showed broad similarities between the ethanol response in each fraction. In addition, we identified many genes that were differentially expressed between the input and IP (TRAP) fractions, highlighting the different makeup of the total mRNA pool vs. translating mRNA pools in a given cell type. Our primary goal in these studies was to identify ethanol-regulated genes in an *in vitro* model that was similar to what we have used in the past using rat cortical astrocytes. By using astrocytes isolated from Aldh1l1-EGFP/Rpl10a mice, we could also identify genes that were differentially translated vs. differentially expressed in a single cell type. These data will be valuable in analyzing our ongoing studies of *in vivo* astrocyte responses to ethanol exposure in Aldh1l1-EGFP/Rpl10a mice. In addition, by leveraging published datasets using the Aldh1l1-EGFP/Rpl10a mice, we can better understand how our i*n vitro* model relates to *in vivo* astrocytes from a developmental time-point perspective. This study also compared our data sets to the *in vivo* astrocyte response to ethanol using published data from *in vivo* astrocytes isolated using a cell-surface antibody-enrichment method following a chronic model of ethanol consumption as well as whole tissue gene expression analysis in the FASD models of ethanol exposure.

In the brain, multiple cell types and heterogeneity within each cell type add complexity to studying the effects of a neurodevelopmental perturbation such as ethanol. Significant progress has been made recently using single-cell and single-nucleus RNA-Seq, but these approaches can be limited by the cost and the ability to detect treatment differences (Ofengeim et al., 2017; Brenner et al., 2020). The use of *in vitro* models has been a valuable tool for understanding complex mechanisms occurring in the brain, allowing the study of individual cell types' responses to alcohol exposure (Goeke et al., 2022). However, there are limitations (Slanzi et al., 2020).

The first question our study addresses is how the total RNA pool and translational RNA pool (mRNA physically associated with ribosomes during translation) differ in a single cell type. We found that approximately half of all detected genes were differentially partitioned between the input and IP fractions (12,313 out of 24,495), with 6,045 genes with higher levels in the IP vs. input and 6,268 genes with higher levels in the input vs. IP. The IP fraction was enriched for genes related to cadherin binding, axon guidance, RNA binding, and VEGFA-VEGFR2 signaling (Supplementary Table 6). Cadherins are cell adhesion molecules that are known to be essential in neuronal development and neurite outgrowth (Tomaselli et al., 1988; Martinez-Garay, 2020). Astrocytes are known to facilitate neuronal development, including axon guidance (Rigby et al., 2020). VEGFA-VEGFR2 signaling is essential for endothelial cell differentiation during angiogenesis, and astrocytes are key facilitators in the process of angiogenesis during early postnatal brain development (Puebla et al., 2022).

It should be noted that the input fraction in the TRAP procedure is a sampling of all RNA present, so it contains both translating RNA and RNA that is not being actively translated. We observed categories related to ribosomes, translation, and protein targeting to the membrane as enriched in the input fraction, suggesting that astrocytes are primed for the translation of these processes and functions. Astrocytes need to respond rapidly to changes in the local environment, and many of the responses elicited in astrocytes occur extracellularly. Therefore, it would be logical to have a pool of translational capacity and membrane-targeting machinery in the form of mRNA that is ready to be translated.

As observed in Figure 2, samples in our data separate most strongly based on the fraction (IP vs. input) in both PCA and hierarchical clustering, but ethanol treatment also clearly separates the samples in the second principal component (PC2) and within each fraction of the hierarchical clustering. Gene pathway and GO enrichment analyses show broad similarities between ethanol-regulated genes from the two fractions (IP and input). Interestingly, the genes downregulated by ethanol appear to be more cohesive in that the enrichment analysis *p*-values are lower and the number of genes in the top enriched categories is larger in both fractions (Figure 3), even though the number of genes that are identified as upregulated vs. downregulated in each fraction is similar (i.e., there is no bias toward one direction of regulation). This indicates that ethanol is upregulating genes in a greater number or a variety of smaller categories, while genes that are downregulated are clumped into a smaller, more concentrated number of categories. Many early transcriptional surveys of gene expression in brain tissue in response to ethanol exposure noted a bias toward downregulated genes (Lewohl et al., 2000; Saito et al., 2002). While we did not observe a bias in the direction of regulation in either of our datasets, the coordinated downregulation caused by ethanol in bulk tissue may reflect a large-scale transcriptional response, while our use of a single cell type allows the identification of the targeted groups of genes that are upregulated and obscured when looking at the bulk tissue with many different cell types and transcriptional responses.

The top GO and pathway categories that were identified in ethanol-downregulated genes in IP and input fractions include broad generic categories such as “RNA binding” and “cell cycle,” as well as the pathway “Myc Targets V1.” Myc-related signaling has been observed in the acute response to ethanol (Kerns et al., 2005) and is differentially expressed in the rodent models of alcohol preferences and avoidance (Sommer et al., 2006). We observed enrichment in lysosome-related genes upregulated by ethanol in both the input and IP fractions (Figure 3). The involvement of lysosomal-related function in the astrocyte response to ethanol has previously been shown. Pla et al. (2016) showed autophagy induction and lysosome enlargement after ethanol treatment in primary astrocyte cultures that were blocked in astrocytes cultured from *Tlr4* knock-out mice, implicating a TLR4-autophagy-lysosome pathway in the astrocyte response to ethanol (Pla et al., 2016). While the TLR4 pathway was not enriched in our ethanol-regulated analyses, *Tlr4* mRNA was upregulated by ethanol in both input and IP fractions (Supplementary Tables 3, 4), and the GO molecular process “regulation of autophagy” is enriched in ethanol-regulated genes from input and IP fractions (Supplementary Table 6).

Our lab is interested in the role astrocytes play in brain development, with a particular emphasis on the extracellular matrix (ECM). During the brain growth spurt, which occurs in the third trimester of human gestation and during the first two postnatal weeks in mice, astrocytes proliferate and provide structural and chemical cues to neurons as they make connections (Clarke and Barres, 2013; Kane and Drew, 2021). In our *in vitro* TRAP data, we noticed that ethanol-upregulated genes were enriched in categories related to axon guidance and chondroitin sulfate-related categories, which provide important extracellular guideposts to developing neurons (Kwok et al., 2012).

One of the challenges in the utilization of *in vitro* models of complex *in vivo* systems is understanding how the *in vitro* model is a good representation of the *in vivo* system and where the models diverge. We utilized both *in vivo* and *in vitro* systems to understand the interaction of astrocytes and neurons in response to ethanol and to model the effects of third-trimester ethanol exposure on brain development. To these ends, we sought to contextualize our *in vitro* data by comparing our translational and transcriptional data to published reports using the same Aldh1l1-EGFP/Rpl10a mouse model we utilized for our cultures. Recent publications studying astrocyte functions in the brain during development and aging allowed us to compare the expression profiles of astrocytes *in vivo* with our *in vitro* data. While examining genes that were identified as altered during development (i.e., the genes that change based on the developmental age of the mice), our *in vitro* data clustered most closely with samples from the cortex of PD1 and PD7 mice (Figure 4). Our astrocytes are isolated from the neocortex of PD0 mice, and we use the cultures to model astrocytes from the early postnatal period of PD7.

We also sought to compare our *in vitro* data to ethanol regulation of gene expression in astrocytes *in vivo*. A method to study cell-type-specific expression profiles involves disruption of the tissue to generate single-cell suspensions and purification of cells by antibody-magnetic bead complexes directed to cell surface proteins. For astrocytes, Acsa2 is commonly used to enrich astrocytes from single-cell suspensions (Erickson et al., 2018; Pan and Wan, 2020). Comparison of our *in vitro* astrocyte data to the medial prefrontal cortex (mPFC) astrocytes following a chronic ethanol exposure paradigm showed many of the same genes as dysregulated by ethanol (Figure 4B), providing support for the relevance of our *in vitro* observations to astrocyte *in vivo* responses.

As our astrocyte cultures are most similar to PD1-PD7 cortical astrocytes, and given our interest in the role astrocytes play during brain development, we also compared our data to recent studies exploring the effects of neonatal ethanol on hippocampal and cerebellar gene expression. We observed a significant overlap in DE and DT genes with both hippocampal and cerebellar ethanol-induced changes. However, compared to the *in vivo* astrocyte comparison above, we observed a greater number of genes with opposite regulation by ethanol in the overlapping genes. There are many potential reasons for the different directions of ethanol regulation, including the presence of multiple cell types in bulk hippocampal or cerebellar RNA, the difference in brain areas examined, differences in the ethanol treatments, the timing of ethanol exposure and when RNA was collected, and the lack of astrocyte-neuron communication in our *in vitro* data, to name a few. However, it is striking that the genes that showed the same direction of regulation by ethanol were enriched in categories related to known astrocyte functions such as cell adhesion, axon guidance, and proteoglycan synthesis and signaling (Wiese et al., 2012; Rigby et al., 2020; Saint-Martin and Goda, 2022). In addition, a comparison of our *in vitro* astrocyte DT and DE genes to a recently published dataset from the hippocampus of C57BL/6J mice administered a single dose of ethanol on PD7 showed that ~90% of genes were regulated in the same direction by ethanol, indicating that our model more closely resembled an acute exposure *in vivo* model (Baker et al., 2022).

Taken together, we found that primary astrocyte cultures isolated from PD0 mice showed the highest similarity in the expression profile of developmentally regulated genes to PD1 and PD7 astrocytes *in vivo* (Figure 4A), indicating that our method of culturing astrocytes is more representative of the early neonatal period than other developmental stages. We observed a significant overlap of DT and DE genes with published reports of ethanol-regulated genes using various models of ethanol exposure. It was somewhat concerning that the direction of ethanol regulation was not always the same. However, it should be noted that some of the comparisons we made were to adult expression profiles after prolonged chronic ethanol exposure (Figure 4B) or in neonatal animals after 24 h of withdrawal from ethanol (Figure 4C). Furthermore, the cellular heterogeneity of intact tissues adds complexity to the interpretation of expression changes as to astrocyte-specific responses to ethanol vs. compensatory changes to alterations in other cell types. Interestingly, the greatest correspondence of our data based on the direction of ethanol regulation was to a recent report in neonatal mice in which expression profiling occurred while alcohol was still in the system (Baker et al., 2022), indicating that our *in vitro* model was most similar to neonatal studies of single ethanol exposures or while ethanol was still present in the system.

## Conclusion

We analyzed the transcriptional and translational pools of primary astrocyte cultures generated from Aldh1l1-EGFP/Rpl10a mice following ethanol treatment to better understand how our *in vitro* model compared to *in vivo* astrocyte responses to ethanol during the early postnatal period in mice. While a large number of differences were observed between the total RNA pool and translating RNA, we observed broad similarities in the GO and pathways enriched in the ethanol-regulated genes in the two fractions. A comparison of our data to recently published reports of developmental astrocyte gene translation showed that our cultures were most similar to PD1 and PD7 cortical astrocytes. In addition, we observed many similarities in our *in vitro* astrocyte response to ethanol to a published report of the *in vivo* astrocyte response to chronic ethanol exposure. Finally, comparing our data to neonatal models of ethanol exposure showed many similarities in genes and pathways that were dysregulated by ethanol.

## Data availability statement

The datasets presented in this study can be found in online repositories. The names of the repository/repositories and accession number(s) can be found in the article/Supplementary material.

## Ethics statement

The animal study was reviewed and approved by VA Portland Health Care System Institutional Animal Care and Use Committee (IACUC).

## Author contributions

MG conceived the project and designed the experiments. JH and XZ contributed to data collection and performed experiments. JH performed the data analysis and wrote the first draft of the manuscript. All authors contributed to the final manuscript.
